# Connection of Dentition in Children with Nursing and Weaning. Clinical Lecture at the Hotel Dieu

**Published:** 1856-04

**Authors:** M. Trousseau


### ARTICLE XVII.

*Connection of Dentition in Children with Nursing and Weaning. Clinical Lecture at the Hotel Dieu.* By M. Trousseau. Translated for the Charleston Medical Journal and Review.

The most elementary questions in medicine are often the least known, and although it might seem unnecessary to pay much attention to matters of apparently so little interest, and which swarm about the practitioner, yet you should remember that Stoll wrote a chapter, entitled "*De quibusdam magni momenti minutiis,*" and accustom yourselves to neglect nothing.

The infant has twenty teeth—the youth twenty-eight—the man thirty-two. The infant's number is not complete until the thirtieth or thirty-sixth month; but they are only temporary teeth, for at the age of seven years he begins to lose them, and to acquire others more durable; at thirteen or fourteen years, this exchange is physiologically accomplished. Except that great king, who was an exception in every thing, and who was born, it is said, with two teeth, the child comes into the world with two unarmed maxillaries, and it is not generally before the eighth month that the first milk teeth make their appearance; but as the laws of nature are somewhat capricious, it not unfrequently happens that one child may have teeth at four months, while another at a year old is still without them; thus no precise limits can be fixed. Generally the two middle inferior incisors appear first, and I always anticipate a troublesome dentition when the superior teeth appear first. These first two teeth always appear together, with an interval between them varying from one to eight days; but *together*, mark it well, and this is the case with these two only. Six or eight weeks after, the two middle superior incisors make their appearance, but not together, and with an interval of eight to thirty days. The process of dentition, then, is rapid with the first two teeth, and slower with the others. Then two more are evolved, the two lateral superior incisors, in a month or two after the superior middle incisors; thus in about a year, the child has six teeth, commencing with two inferior, and continuing with four superior.

The teeth of infants come out in *groups, dentes in infantibus catervatim erumpunt.* The first group consists of two middle inferior incisors at about eight months; second group, two middle superior incisors about ten months; third group, two lateral superior incisors at about a year; fourth group, two lateral inferior incisors and the first four molars (six teeth in this group) at fourteen to sixteen months; fifth group, four cuspids at eighteen months to two years; sixth group, four second and last molars at thirty or thirty-six months.

The cuspids appear after the child has completed twelve teeth, and is eighteen months or two years old; their evolution occupies about two or three months. There is then an interval of six to ten months; and at the age of three years, when it has brought out its last group, the process of dentition is over.

I have not spoken of groups undesignedly; for you will see that, in connection with weaning, it requires attention. An important fact is that, after the appearance of each group, the child has a short period of repose; and this is the propitious occasion for weaning. Generally no regard is paid to this; infants are weaned indiscriminately, when they have two, seven, nine, eleven, or fourteen teeth. But you most observe closely; otherwise you will lose your little patients by that terrible affection of the bowels, cholera infantum.

You will, doubtless, be often consulted about the time for weaning; never decide until you have closely examined the circumstances of dentition, and do not authorize the mother to wean her child until it has six, twelve, or sixteen teeth. In the best practice, weaning should never begin in the interval after the evolution of the first group; the subject is too young, and is usually only eight months old. And it is only with very great caution, that you should begin after the third group—if strongly desired by the parents you may consent; for there is a space of a month or six weeks of repose before the coming out of the fourth group; but be careful to remember that the child has only six teeth, that it is only a year old, and that foreign nourishment will not always answer your expectation.

The most favorable time for weaning is, undoubtedly, the interval which separates the fourth from the group; at that epoch, the child is supplied with twelve teeth, eight incisors and four molars, and has before it a sufficiently long period for repose, about two months, during which there is nothing to apprehend from the bowels; thus when the cuspids begin to appear, (and the evolution of this group is the most dangerous,) it is habituated to its new diet, and prepared for the crisis which awaits it.

Wait, then, for the fourth group before weaning. If the health of the mother, or nurse, or family interests, oblige you to authorize premature weaning, do so when there are six teeth; but if not forced to yield to some such consideration, defer until there are twelve.

Do not suppose that matters will, in all cases, progress thus regularly. You will probably see some children cut their molars before their incisors, or the superior incisors before the inferior; for although dentition proceeds ordinarily as I have indicated, yet there are often irregularities which are very embarrassing to the physician, whose chief desire is to ascertain some interval of repose. Do the best you can; examine the state of the gums and wean as soon as possible after the evolution of a tooth, as there will then probably be a brief respite.

Among the accidents to which the period of dentition is liable, the gravest and most tenacious are those of the alimentary canal. Several days before hand, the child is restless, it sleeps little, utters violent cries, sucks its fingers, presses the nipple of the nurse, refuses to take any supplementary nourishment, and sometimes even to nurse; the gums are red, and there exists a very perceptible elevation at the point at which the teeth are to appear; it coughs, the voice changes, the mucous membrane of the mouth is irritated; but as soon as the child has two teeth, the surrounding gingival tissue will become very red and tumefied.

If you administer mercury to an individual who has not a tooth in his head, and who wears false teeth, neither salivation, nor mercurial stomatitis, will ensue; but should this person have a single tooth remaining, mercurial manifestations will take place around it; the surrounding tissue of that tooth will be inflamed, while other portions will not be affected by the inflammation. Now the case is the same for the first teeth of the infant; their issue has no effect upon the gums; but at the evolution of the second group, the gums redden and swell.

With nearly all children, the process of dentition is accompanied with diarrhea; moderate sometimes, but often intense, with evacuations of a greenish color, and resembling a hash of green herbs, or clots of curdled milk, containing glairy, and even sanguineous matter. In certain cases, there is much tenesmus, with prolapsus of the rectum. These accidents, which precede by several days the issue of the tooth, often continue until the whole group is cut. Should the diarrhea not cease, you will resort to all the necessary means to restrain and ameliorate it. During this diarrhea, weaning should not be permitted, unless the milk of the nurse should appear to you to contribute to the internal flux.

In the summer season, the disorders attending dentition are generally of the intestines, rarely of the respiratory passages. Intestinal derangements, fever, peripneumonic catarrh, and other pathologic manifestations concerning the lungs, are of frequent occurrence in the winter.

I must warn you against a certain vulgar prejudice, and enjoin upon you to combat it whenever presented. You will hear it said and repeated that *diarrhea is good for children:* it is not so, and it will often cause the death of your little patients. Diarrhea brings on chronic enteritis, and this debilitates and carries off children. By combating, therefore, this intestinal flux, you will guard the better against affections in other parts.

It is also considered improper to remove the scurf which frequently covers children's heads; but this ridiculous prejudice has ceased in England and in the United States; let us disregard it here.

If, during dentition, the evacuations should become somewhat more easy, without degenerating into diarrhea, you need not fear it; but should it continue several days, it must be checked.

The opinion is current, that constipated children are often attacked by convulsions, while those having diarrhea are never thus affected. This is not the case; diarrhea is a frequent source of convulsions, and a healthy state of the bowels generally prevents them.

I call your attention particularly to alimentation, for it is a matter of the utmost importance. Indeed, if you neglect it, diarrhea will ensue, and successively enteritis, serious indigestion and convulsions. Nothing is more common than indigestion, induced by enteritis, and leading to convulsions, and nothing is more calculated to alarm parents; they generally lose all self-command; and while the servants or the neighbors hurry off for a physician, the mother, under the advice of some over-officious old woman, pours boiling water over the feet and hands of her child, and scalds it often to death. And this reminds me of what once occurred to an eminent colleague, Professor Marjolin, during the course of a typhoid fever, which had thrown him into a profound stupor. They applied to his legs towels dipped in water at 190°, causing large eschars, which were not healed for several months.

With regard to convulsions, the less you do the better; indeed, the paroxysm is generally over before you arrive; and although the symptoms should reappear two or three times, yet by the next day every thing is usually over. If there has been any indigestion, administer a laxative, for it is best to get rid of undigested food; let the child nurse very little; give it mucilaginous drinks, have it bathed, if necessary, and you will soon cause the disappearance of all alarming symptoms. In general, every thing succeeds, even the infinitesimals of the infinitely ridiculous homœopathy.—*Gazette des Hospitaux, Dec. 11th*, 1855.

